# Successful Establishment of Plasmids R1 and pMV158 in a New Host Requires the Relief of the Transcriptional Repression of Their Essential *rep* Genes

**DOI:** 10.3389/fmicb.2017.02367

**Published:** 2017-12-01

**Authors:** José Á. Ruiz-Masó, Luis M. Luengo, Inmaculada Moreno-Córdoba, Ramón Díaz-Orejas, Gloria del Solar

**Affiliations:** Molecular Microbiology and Infection Biology Department, Centro de Investigaciones Biológicas, Consejo Superior de Investigaciones Científicas, Madrid, Spain

**Keywords:** plasmid repopulation, establishment phase replication, R1 replicon, pMV158 replicon, Cop transcriptional repressors, plasmid replication rate

## Abstract

Although differing in size, encoded traits, host range, and replication mechanism, both narrow-host-range theta-type conjugative enterobacterial plasmid R1 and promiscuous rolling-circle-type mobilizable streptococcal plasmid pMV158 encode a transcriptional repressor protein, namely CopB in R1 and CopG in pMV158, involved in replication control. The gene encoding CopB or CopG is cotranscribed with a downstream gene that encodes the replication initiator Rep protein of the corresponding plasmid. However, whereas CopG is an auto-repressor that inhibits transcription of the entire *copG-repB* operon, CopB is expressed constitutively and represses a second, downstream promoter that directs transcription of *repA*. As a consequence of the distinct regulatory pathways implied by CopB and CopG, these repressor proteins play a different role in control of plasmid replication during the steady state: while CopB has an auxiliary role by keeping repressed the regulated promoter whenever the plasmid copy number is above a low threshold, CopG plays a primary role by acting coordinately with RNAII. Here, we have studied the role of the regulatory circuit mediated by these transcriptional repressors during the establishment of these two plasmids in a new host cell, and found that excess Cop repressor molecules in the recipient cell result in a severe decrease in the frequency and/or the velocity of appearance of transformant colonies for the cognate plasmid but not for unrelated plasmids. Using the pMV158 replicon as a model system, together with highly sensitive real-time qPCR and inverse PCR methods, we have also analyzed the effect of CopG on the kinetics of repopulation of the plasmid in *Streptococcus pneumoniae*. We show that, whereas in the absence of CopG pMV158 repopulation occurs mainly during the first 45 min following plasmid transfer, the presence of the transcriptional repressor in the recipient cell severely impairs the replicon repopulation and makes the plasmid replicate at approximately the same rate as the chromosome at any time after transformation, which results in maximal plasmid loss rate in the absence of selection. Overall, these findings indicate that unrepressed activity of the Cop-regulated promoter is crucial for the successful colonization of the recipient bacterial cells by the plasmid.

## Introduction

Plasmids specify replication control systems that enable them to maintain a characteristic steady-state concentration (copy number) in their host cell. These regulatory systems are *trans*-acting and can sense and correct stochastic up and down fluctuations of the plasmid copy number in individual cells. By adjusting the replication initiation rate in response to changes in the intracellular plasmid concentration, control systems manage to keep the steady-state condition, where every plasmid copy replicates, on average, once per cell generation (Nordström, 1990). Steady-state control of plasmid replication has been analyzed at a deep level and a variety of different regulatory circuits have been mechanistically characterized (del Solar and Espinosa, 2000; Das et al., 2005; Nordström, 2006). Antisense RNA (asRNA)-mediated control of plasmid replication is widely spread in theta- and rolling circle-replicating plasmids from Gram-positive and Gram-negative bacteria (del Solar and Espinosa, 2000; Brantl, 2014). The small regulatory RNAs involved in control of plasmid replication and copy number are *bona fide* (i.e., *cis*-encoded) asRNAs, as they are encoded on the DNA strand opposite to an RNA essential for replication initiation, namely a pre-primer or an mRNA for the replication initiator protein (Rep). These asRNAs base-pair to their target (sense) RNA to inhibit its function and/or the completion of its synthesis through a variety of mechanisms, including inhibition of primer maturation, transcription attenuation, prevention of formation of a translation activator RNA pseudoknot, and inhibition of translation of either *rep* or a leader-peptide reading frame to which *rep* is translationally coupled (del Solar and Espinosa, 2000; Brantl, 2014). Most frequently, asRNAs controlling plasmid replication are metabolically unstable, *trans*-acting inhibitory elements, whose synthesis is directed by unregulated and strong promoters. These features enable them to sense and correct rapidly up and down fluctuations of the plasmid copy number in individual cells (del Solar and Espinosa, 2000; Wagner et al., 2002; Brantl, 2014).

Although the asRNA is the sole replication control element in some plasmids (pT181 family, IncB/IncIα family, ColE2), it is accompanied, in others, by a regulatory protein that acts either as a transcriptional repressor (Cop proteins in R1, Inc18, and pMV158 families) or as an RNA-binding protein (Rom/Rop protein in ColE1-like plasmids) (del Solar and Espinosa, 2000; Brantl, 2014). Rom/Rop and CopB of ColE1- and R1-like replicons, respectively, have been largely considered as mere auxiliary elements because of their rather secondary role in the steady-state plasmid replication control, when the activity of these proteins is almost saturating (Nordström et al., 1984; Rosenfeld and Grover, 1993; Atlung et al., 1999; Summers, 2009). In contrast, efficient replication control of plasmids of the Inc18 and pMV158 families requires the coordinated participation of the asRNA and of the transcriptional repressor Cop protein, both elements playing a primary regulatory role (del Solar and Espinosa, 2000; Brantl, 2014).

**Figure 1:**
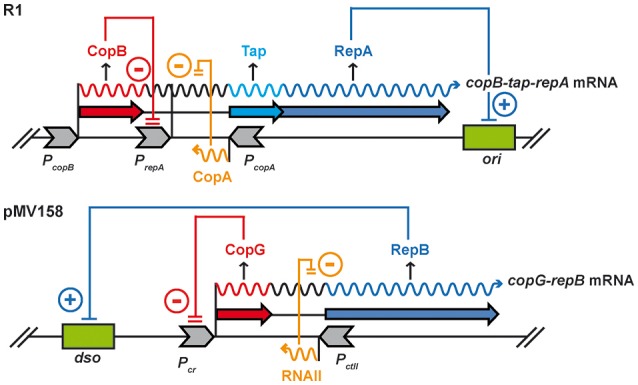
Schematic comparison of the mechanisms controlling expression of the essential *rep* gene in R1 and pMV158. In R1, *repA* is transcribed from *P*_*copB*_ and *P*_*repA*_ promoters, and CopB represses transcription from the stronger *P*_*repA*_ promoter. Gene *copA*, which encodes an asRNA, overlaps the intergenic region of the *copB-tap-repA* operon. CopA exerts an indirect control on *repA* translation by inhibiting synthesis of Tap protein, to which RepA synthesis is coupled. In pMV158, the *copG-repB* operon is transcribed from the *P*_*cr*_ promoter, which is negatively regulated by transcriptional repressor CopG. asRNAII, transcribed from *P*_*ctII*_ promoter, binds to its complementary sequence in the *copG-repB* mRNA, thus inhibiting translation of the replication initiator *repB* gene.

Plasmid R1, originally isolated from *Salmonella enterica* serovar Paratyphi, is a low-copy-number, multiresistance, conjugative plasmid of the IncFII incompatibility group. It has a narrow host-range restricted to the *Enterobacteriaceae* family. The R1 elements and circuits involved in steady-state plasmid replication control and maintenance have been studied in great detail (Nordström et al., 1984; Olsson et al., 2004; Nordström, 2006). In addition to the origin of replication (*oriR1*), the R1 basic replicon includes the *repA* gene for the replication initiator protein and the two replication control genes, *copB* and *copA*, encoding, respectively, the transcriptional repressor CopB protein and the CopA asRNA (Figure 1). The RepA protein is rate limiting for initiation of replication. The essential *repA* gene is transcribed from two promoters, namely *P*_*copB*_ and *P*_*repA*_. The upstream *P*_*copB*_ promoter directs constitutive transcription of *copB, tap* (the leader peptide reading frame) and *repA*, which is translationally coupled to *tap*. Transcription from the downstream CopB-regulated *P*_*repA*_ promoter gives rise to the shorter bicistronic *tap-repA* mRNA (Figure 1). When unrepressed, the *P*_*repA*_ promoter is about twice as strong as *P*_*copB*_, although under normal conditions during the steady-state plasmid replication, *P*_*repA*_ is almost totally (90%) switched off by CopB–mediated repression (Olsson et al., 2004). Since CopB acts as a tetramer (Riise and Molin, 1986), the activity of *P*_*repA*_ is likely to be strongly dependent on plasmid concentration. It has been shown that the presence of extra copies of *copB in trans*, which further reduces the already low activity of *P*_*repA*_, increases 7-fold the rate of loss of a Par^+^ derivative of the R1 basic replicon (Olsson et al., 2004). The steady-state activity of *P*_*repA*_ is thought to stabilize the plasmid inheritance both by speeding up R1 replication in cells with very few plasmid copies (thus decreasing the frequency of these cells) and by slightly increasing the average plasmid concentration. CopB also plays a main role in the coupling between the *kis-kid* auxiliary maintenance system and the basic replicon of the plasmid (López-Villarejo et al., 2015). The switch of this coupling is the antitoxin Kis, whose levels decrease in cells with lower-than-average plasmid copy number. Decrease of Kis concentration activates the Kid toxin, which is an RNase with two efficient target sites in the intergenic region of the *copB-repA* mRNA (Pimentel et al., 2005). Cleavage at these sites reduces the CopB levels, which leads to activation of *P*_*repA*_ and subsequent increase in plasmid replication efficiency. Hence, the *kis-kid* system coupled to the CopB-mediated loop functions as a safety device when the plasmid copy number is very low.

Promiscuous plasmid pMV158 was originally isolated from a clinical strain of *Streptococcus agalactiae* (Burdett, 1980) and subsequently transferred to a large number of bacterial genera and species. The detailed analysis of its replicon has made pMV158 the prototype of a vast family of rolling circle-replicating plasmids (Ruiz-Masó et al., 2015; Boer et al., 2016). The pMV158 basic replicon includes a compact region containing the double-strand origin (*dso*) (Ruiz-Masó et al., 2007) as well as the genes that encode the replication initiator protein (RepB) (Ruiz-Masó et al., 2004; Boer et al., 2009) and the two replication control elements (transcriptional repressor CopG and asRNA RNAII) (del Solar et al., 1995; Gomis-Rüth et al., 1998; Hernández-Arriaga et al., 2009; López-Aguilar et al., 2015) (Figure 1). Like the *rep* gene product of R1, RepB of plasmid pMV158 is the rate-limiting factor for the replication initiation process. Unlike R1, the streptococcal plasmid expresses the essential *rep* gene from a single promoter (*P*_*cr*_), which directs cotranscription of the *copG-repB* operon and is subjected to CopG-mediated regulation (del Solar et al., 1990) (Figure 1). In the unrepressed state, *P*_*cr*_ seems to be even stronger than promoter *P*_*ctII*_ that directs synthesis of countertranscript RNAII, a situation that contrasts with the plasmid replication control systems based exclusively on asRNA, where the transcription rate of the essential RNA is constant but rather low compared with that of the asRNA (del Solar and Espinosa, 2000). Unsuccessful repression of promoter *P*_*cr*_ in pMV158 derivatives encoding a defective CopG repressor leads to a 5-fold increase in the plasmid copy number (del Solar et al., 1990, 1995). On the other hand, the presence *in trans* of high dosages of the autoregulated *copG* gene has been shown to decrease by ~35% the steady-state copy number of the pMV158 replicon in *S. pneumoniae* (del Solar et al., 1995).

Despite the quite deep current knowledge about the involvement of the Cop transcriptional repressors in the control of the steady-state plasmid replication, very little has been reported so far on their role in the establishment phase replication. The Cop regulatory elements have been proposed to play an important role during plasmid establishment in a new bacterium based on the fact that the Cop-regulated promoter, when unrepressed, determines high transcription rates of the essential *rep* gene (del Solar et al., 1990, 1995; del Solar and Espinosa, 2000; Olsson et al., 2004; Brantl, 2014). Yet, only non-published results have been invoked in a few articles to suggest the involvement of the Cop regulatory loops of pMV158 and R1 in the establishment phase replication of these plasmids (Nordström and Nordström, 1985; del Solar and Espinosa, 2000; Olsson et al., 2004).

In this work, we have analyzed the effect of the Cop proteins of R1 and pMV158 on the establishment of these plasmids. To this end, we have electrotransferred a mini-R1 derivative (pKN1562) to *Escherichia coli* and *Salmonella* Typhimurium (*S*. Typhimurium hereafter) cells that either contain or lack a compatible recombinant plasmid encoding CopB. Similarly, we have transferred pLS1 (a *mob*^−^ derivative of pMV158, Lacks et al., 1986) to naturally-competent pneumococcal cells and to electrocompetent cells of *Staphylococcus aureus* either containing or lacking a compatible recombinant plasmid encoding CopG. We show that, irrespective of the system employed, the presence of these proteins in the recipient cell selectively impairs the establishment of the cognate plasmid, resulting in a decrease in the frequency of total or early transformant colonies. By using the pneumococcal host as a model system, we also show that repopulation of the pMV158 replicon is almost abolished when autoregulated *copG* is supplied *in trans* at a high gene dosage.

## Materials and methods

### Bacterial strains and plasmids

Bacterial strains employed, and their uses for this work, are summarized in Table 1. Plasmid constructions used throughout this study, as well as their relevant features, are listed in Table 2. *S. pneumoniae* 708 was the host for pLS1, pLS1*cop7*, pC194, pCGA3, pCGA3n, pCGA30, and pAMβ1 plasmids. Pneumococcal cells were grown at 37°C in AGCH medium (Lacks et al., 1986) supplemented with 0.3% sucrose and 0.2% yeast extract. *S. aureus* RN4220 was the host for pLS1, pT181*cop608*, pC194, pCGA3, pCGA3n, and pCGA30 plasmids. Staphylococcal cells were grown at 37°C in brain heart infusion medium (BHI, Difco). *E. coli* C600 and *S. Typhimurium* SL1344 were the hosts for pUC18-*copB*, pKN1562, and pACYC184 plasmids; cells were grown at 30°C in Lysogeny broth (LB) medium.

**Table 1:** Strains used in this study.

**Bacteria**		
**Bacteria**	**Characteristics**	**Source**
*Escherichia coli* C600	K12 derivate	Appleyard, 1954
*Salmonella* Typhimurium SL1344	Wild-type strain	Hoiseth and Stocker, 1981 NC_016810.1
*Staphylococcus aureus* RN4220	Restriction-deficient mutant of strain 8325-4	Kreiswirth et al., 1983
*Streptococcus pneumoniae* 708	*end*-1 *exo*-1 *trt*-1 *hex*-4 *mal*M594	Lacks and Greenberg, 1977

**Table 2:** Plasmids used in this study.

**Plasmids**				
**Plasmids**	**Characteristics**	**Antibiotic marker**	**Copy number**	**Source**
pACYC184	Plasmid vector containing the minimal p15A replicon	Cm^R^ Tc^R^	≈15^a^	Chang and Cohen, 1978
pAMβ1	Conjugative plasmid from *Enterococcus faecalis* DS5	Em^R^	≈7^c^	Leblanc and Lee, 1984
pCGA3	*copG* cloned in the high copy number vector pC194	Cm^R^	≈200^e^	del Solar et al., 1995
pCGA3n	*copG* cloned in the medium copy number vector pC194n	Cm^R^	≈25^e^	del Solar et al., 1995
pCGA30	pCGA3 derivative with a 48-bp deletion that includes the−10 sequence of the *P_*c*_*_r_ promoter, and the *SD* sequence and the two first codons of *copG*.	Cm^R^	≈200^e^	del Solar et al., 1993
pC194	Rolling circle replicating plasmid from *S. aureus*	Cm^R^	≈200^e^	del Solar et al., 1995
pKN1562	Wild-type mini-R1 plasmid carrying the wt kis-kid system	Km^R^	1–2	Molin et al., 1979
pLS1	Non-mobilizable pMV158 derivative plasmid (Δ*mobM*, Δ*ssoU*)	Tc^R^	46.0 ± 5.0^c^^,^^d^	Stassi et al., 1981
pLS1*cop7*	pLS1 copy-up mutant	Tc^R^	182.1 ± 15.5^c^^,^^d^	del Solar et al., 1990
pT181-*cop608*	pT181-based high copy number mutant plasmid	Tc^R^	800–1,000^b^	Rasooly et al., 1994
pUC18- *copB*	*copB* cloned in the high copy number vector pUC18	Amp^R^	≈500^a^	López-Villarejo et al., 2015

a *Plasmid copy number (PCN) determined in E. coli C600*,

b *S. aureus*,

c *and S. pneumoniae*,

d *PCN determined by RT-qPCR in this work (± standard error)*,

e *Approximate PCN value estimated by comparative quantitation of total DNA extracted from pneumococcal strains containing the target plasmid or the control pLS1 plasmid; PCN of the latter has been determined by RT-qPCR*.

### Plasmidic and genomic DNA preparations

Plasmidic DNA (pDNA) content from pneumococcal transformants was analyzed by preparing total DNA crude extracts as described (del Solar et al., 1987). These DNA preparations were also used to estimate the relative plasmid copy number from the ratio between the intensities of the plasmid and chromosome DNA bands quantified for the plasmid of interest relative to a plasmid control whose copy number has been precisely determined, after correcting for the difference in size of both plasmids (del Solar et al., 1995). Plasmids pLS1, pLS1*cop7*, pC194, pCGA3, pCGA3n, and pCGA30 were isolated from *S. pneumoniae* 708 and purified by two consecutive CsCl/ethidium bromide density gradient centrifugations, as described (Lacks et al., 1986). Plasmid pAMβ1 was isolated from *S. pneumoniae* 708 and purified by alkaline lysis as described (Stassi et al., 1981). Plasmid pT181*cop608*, isolated from *S. aureus* RN4220, and plasmids pKN1562, pUC18*-copB*, and pACYC184, isolated from *E. coli* C600, were purified using a Jetstar Plasmid Midiprep Kit (Genomed). pDNA content from staphylococcal transformants was analyzed by the same alkaline lysis method used for *S. pneumoniae*. In both midipreps and alkaline lysis procedures lysostaphin (50 μg/ml) was added to the cell resuspension buffer in order to facilitate staphylococcal cells lysis.

Genomic DNA (gDNA) used as template for real-time quantitative PCR (qPCR) and inverse PCR (iPCR) was isolated from pneumococcal cultures in exponential growth phase, which was determined by measurement of optical density at 650 nm. The DNA was extracted from cells of *S. pneumoniae* 708 with different plasmid content by using the Wizard® Genomic DNA Purification Kit (Promega) optimized for *S. pneumoniae*. Cells resuspended in 50 mM EDTA were incubated with 0.04% of deoxycholate and 0.1 mg/ml of Proteinase K for 10 min at 37°C. Next, and before proceeding with the lysis step, the cellular suspension was quickly frozen on a mixture of dry ice and ethanol and stored at −80°C. With this method, aliquots taken at different time intervals were processed simultaneously from the lysis step. Moreover, 0.05 μg/ml of glycogen (molecular biology grade) was added in the isopropanol precipitation step to facilitate gDNA recovery from diluted samples. Contrarily to the samples where no glycogen was added, the gDNA yield of the samples treated with glycogen was found to be nearly proportional to the total amount of lysed cells. Concentration of the gDNA was determined with a Qubit fluorometer by using the Qubit HS dsDNA Assay Kit (Molecular Probes).

Purified gDNA was digested with EcoRI, a restriction enzyme that linearizes the pLS1 DNA but leaves intact the plasmidic and chromosomal amplicons (i.e., the DNA segments to be amplified in the qPCR assays). This method has been developed to obtain accurate qPCR-based copy number results for plasmids (Providenti et al., 2006).

### Calculation of the experimental plasmid loss rate

The experimental loss rate (*L*_*ex*_) of pLS1 and pLS1*cop7* in newly transformed pneumococcal cells was calculated from the equation (Gerdes et al., 1985):

(1) $$\begin{array}{r} T/T_{0}={\left(1-L_{ex}\right)}^{n}, \end{array}$$

where *T*_0_ and *T* are, respectively, the fractions of transformants *ab initio* and after *n* generations. This equation can be converted into a linear function by taking logarithms,

(2) $$\begin{array}{r} \log\left(T/T_{0}\right)=\log\left(1-L_{ex}\right)n, \end{array}$$

where log(1-*L*_*ex*_) is the slope of the linear regression fit in the plot of the experimental values of log(*T/T*_0_) against the number of cell generations (*n*).

### Transformation of bacterial species with plasmid DNA

Transformation of *E. coli* C600 and *S*. Typhimurium SL1344 cells was performed by electroporation essentially as described (Dower et al., 1988). Competent cultures of *E. coli* C600 and *S*. Typhimurium SL1344 and those of the same strains harboring pUC18-*copB* as the resident plasmid were transformed with 0.2 μg of plasmid DNA of pKN1562 or pACYC184. Transformants were grown on LB-agar plates with antibiotic selection according to the resistance carried by the plasmids: 50 μg/ml of kanamycin (Km) for pKN1562, 50 μg/ml of ampicillin (Amp) for pUC18-*copB*, or 20 μg/ml of chloramphenicol (Cm) for pACYC184. Competent cells of *S. aureus* RN4220 were prepared and transformed by electroporation following the procedure depicted in Augustin and Götz (1990). Competent staphylococcal cultures (50 μl) harboring pC194, pCGA3, pCGA3n, or pCGA30 as the resident plasmid were transformed with 0.5 μg of DNA of the donor plasmid (pLS1 or pT181). After allowing for phenotypic expression (60 min), all cultures were treated for 30 min with 0.5 μg/ml of tetracycline (Tc), a sub-inhibitory concentration of the antibiotic that allows induction of the pT181 *tet* gene. Cells transformed with pLS1 or pT181 were selected on BHI-agar plates containing 5 μg/ml of Tc, for selection of the entering plasmid, and 3 μg/ml of Cm, for resident plasmid selection.

Competent cells of *S. pneumoniae* 708 were prepared and transformed as described (López et al., 1982). Three independent lots of naturally competent cells were prepared from each of the four different strains harboring pC194, pCGA3, pCGA3n, or pCGA30 as the resident plasmid. Since the development of pneumococcal competence is influenced by many factors (Attaiech et al., 2015), including the exact composition of the semi-defined AGCH culture medium, the 12 competent cultures were each tested for their level of competence by transformation with chromosomal DNA from a strain able to grow in maltose. Although the transformation efficiency for maltose utilization varied significantly from lot to lot of competent cells of the same strain, the level of competence for chromosomal transformation of the competent cells prepared in parallel was, consistently, 1.5 to 2-, 2 to 3-, and 4 to 5-fold higher for the strains containing pCGA30, pCGA3n, and pCGA3, respectively, than for the strain harboring pC194. The reason for the apparent increase in the natural competence of *S. pneumoniae* when the DNA of gene *copG* is present has not been investigated yet. Cultures (1 ml) of competent pneumococcal cells were transformed with 0.25 μg of DNA of the donor plasmid (pLS1 or pAMβ1). After allowing for phenotypic expression (70 min), cultures were induced with 0.5 μg/ml of Cm for 20 min. Transformants were selected using agar plates containing 1 μg/ml of Tc for pLS1 or 1 μg/ml of erythromycin for pAMβ1. In these plates, selection for the resident plasmid (3 μg/ml of Cm) was maintained. Since pneumococci grow best when protected from air, the basal AGCH-agar layer containing the cells was overlaid with AGCH-agar medium.

### Repopulation kinetics assays

For repopulation kinetics experiments, cultures of competent pneumococcal cells harboring pCGA3 and pCGA30 were subjected to a modified version of the transformation procedure described in López et al. (1982) that yielded 10-fold higher competence levels. Competent cultures (OD_650_ = 0.3) were diluted 1/20 in 10 ml of AGCH medium supplemented with 0.3% of sucrose, 0.001% of CaCl_2_ and a sub-inhibitory concentration of Cm (0.5 μg/ml), in order to keep the induced expression of the *cat* gene. The cells were cultured at 37°C to an OD_650_ of 0.3, and the cultures were cooled to 30°C for 15 min. Then, the cells were transformed with 2 μg of DNA of the pMV158 derivative (pLS1 or pLS1*cop7*) by incubation for 30 more min at the same temperature. To stop the transformation process, pancreatic DNase I was added to a final concentration of 2 μg/ml, and the incubation at 30°C was prolonged for 20 more min. Next, the cultures were diluted 1/10 in pre-warmed (37°C) AGCH medium supplemented with 0.3% sucrose, 0.2% yeast extract and 0.5 μg/ml of Cm, and incubated at 37°C up to 150 min. Immediately after dilution, and at the indicated time intervals, 10 and 0.1-ml aliquots of the cultures were withdrawn and used, respectively, to extract the gDNA and to determine the number of total viable cells (c.f.u./ml) and the fraction of transformants. Transformants were selected in three-layered AGCH-medium agar plates containing 3 μg/ml of Cm and 1 μg/ml of Tc. Cells were deposited in the basal layer that was overlaid with a second layer of AGCH-agar medium. The plates were then incubated at 37°C for 2 h before antibiotics for selection were included in the third layer and spread across the rest of the plate by diffusion.

No-transformation control experiments were also performed to ensure that plasmid DNA amplified by real-time qPCR arose from the transformed cells and was not contaminant DNA that escaped from the DNase I digestion. For this purpose, we followed the same transformation procedure as described above but adding simultaneously 2 μg of pLS1*cop7* DNA and DNase I (2 μg/ml), in order to avoid transformation. Aliquots of 10 ml of the no-transformed cultures were taken immediately after dilution and after 30 min of incubation at 37°C. These cell aliquots were processed as described to obtain gDNA. In all cases, before proceeding with the gDNA isolation protocol, cells were washed with 10 ml of 1X PBS (phosphate-buffered saline).

The total number of cell generations (*n*) was calculated according to the following equation:

(3) $$\begin{array}{r} n=log\left(V/V_{0}\right)/log2, \end{array}$$

where *V* is the number of viable cells (c.f.u.) at any of the times analyzed and *V*_0_ is the initial value of viable cells.

A similar expression was used to calculate the number of gDNA duplications (*D*_C_) at a given time interval (*ti-tj*):

(4) $$\begin{array}{r} D_{c_{i-j}}=log\left(gDNA_{j}/gDNA_{i}\right)/log2, \end{array}$$

where *gDNA*_*j*_ and *gDNA*_*i*_ are the amounts (ng) of gDNA obtained (after precipitation in the presence of glycogen, see above) at the times *tj* and *ti*, respectively.

### Determination of the copy number of a specific pMV158 amplicon relative to a chromosomal amplicon in transformed pneumococcal cells by qPCR

Two primer sets specific to the PcrA helicase single-copy reference gene (*pcrA*) of *S. pneumoniae R6* (Hoskins et al., 2001) and to the tetracycline resistance TetL protein gene (*tetL*) of pMV158 were designed. Oligonucleotide primers sets (Table 3) were designed with Primer3 v0.4.0 (Koressaar and Remm, 2007; Untergasser et al., 2012) based on the pLS1 sequence (NC_010096.1) and on the *S. pneumoniae* R6 (NC_003098.1) *pcrA* sequence. Criteria used during primer design were that primers had predicted Tm of ~59°C and that they generated amplicons ~140 bp in length.

**Table 3:** Oligonucleotides used in this study.

**Target**	**Accession No**.	**Sequence (5′-3′)^a^**	**Length (nt)**	**Primer position**	**Product size (bp)**
**Sequence of primers used for real-time qPCR**					
*pcrA*	NC_003098.1	F: GAGTTGGTTGAGTCCGTCCT	20	980570–980589	144
		R: TGTCACATCCGTGGTGTCAT	20	980713–980694	
*tetL*	NC_010096.1	F: TGCGAGTACAAACTGGGTGA	20	1879–1898	146
		R: ACCCAATTACCGACCCGAAA	20	2024–2005	
**Sequence of primers used for iPCR**					
*del-5*	NC_010096.1	F: GTTTGAGGCTCGTCAAATC	19	514–532	4403
*dso2*	NC_010096.1	R: CAGCTCTAAGGCTAAAGGCG	20	508–489	

a *F and R indicate forward and reverse primers, respectively*.

qPCRs were conducted in a total volume of 20 μl using a LightCycler® 96 real-time detection system (Roche) and the FastStart Essential DNA Green Master (Roche), as per manufacturer's recommendations. Decimally diluted EcoRI-digested gDNA preparations (14, 1.4, 0.14 ng per reaction) were analyzed using 0.5 μM (final concentration) of the specific forward and reverse primers of either primer-pair used (Table 3). To prepare the reactions and minimize pipetting errors 2 μl of template DNA were added to individual qPCRs. Thermal cycling conditions were as follows: initial denaturation at 95°C for 5 min, followed by 40 cycles of 95°C for 10 s (denaturation), 59°C for 30 s (primer annealing), and 72°C for 20 s (elongation). A melting curve analysis of the PCR products, with a temperature gradient of 0.1°C/s from 59 to 95°C, was performed to confirm the purity and specificity of the PCR products. Two independent qPCR trials were conducted for each template source. In each trial, triplicate samples of the three different amounts of template were analyzed. Control samples without template DNA were also analyzed.

Relative copy number (*CN*) of the pMV158 amplicon was calculated using equation:

(5) $$\begin{array}{r} CN={\left(1+\;E_{pcrA}\right)}^{Ct_{pcrA}}/{\left(1+\;E_{tetL}\right)}^{Ct_{tetL}}, \end{array}$$

where *E*_*pcrA*_ and *E*_*tetL*_ are, respectively, the PCR amplification efficiencies of the chromosomal and plasmid amplicons, and *Ct*_*pcrA*_ and *Ct*_*tetL*_ are the mean threshold cycle values obtained for the corresponding amplicons. A CN value was calculated for each of the three template concentrations analyzed, and the mean and standard deviation of the six values (two independent trials with three different template concentrations each) were determined.

*E* values of target (*E*_*tetL*_) and reference (*E*_*pcrA*_) sequences were empirically calculated for each qPCR trial. For that purpose, mean *Ct* values were plotted against the logarithm of the amount of total DNA template in the assay. From the slope of the curve generated by linear regression of the plotted points, the PCR amplification efficiency was determined according to the equation:

(6) $$\begin{array}{r} E=\;10^{-1/slope}-1, \end{array}$$

Although the *E* values for both amplicons were higher than 0.9, we have chosen Equation (5) to calculate the relative copy number of the plasmid amplicon as it allows taking into account the slight differences between *E*_*target*_ and *E*_*reference*_ that we have observed.

### Determination of the relative amount of circular plasmid DNA in transformed pneumococcal cells by iPCR

The plasmidic DNA (pDNA) present in the gDNA isolated from the transformed pneumococcal cells was used as template to perform an inverse PCR protocol with a primer set of divergent oligonucleotides. iPCR was performed using the Phusion High Fidelity (HF) (Thermo Scientific) DNA polymerase. Amplification reactions (20 μl) contained 0.7 ng of gDNA and 0.5 μM of the specific forward and reverse primers (Table 3). Thermal cycling conditions comprised 25 cycles (98°C for 10 s, 59.5°C for 30 s, and 72°C for 1 min and 25 s) plus a final extension step of 10 min at 72°C. The amplification reaction yielded a linear dsDNA fragment corresponding to almost the entire pLS1*cop7* plasmid. The products of iPCR were analyzed on 0.8% agarose gels, stained with GelRed (Biotium), and quantified with the aid of a Gel Doc (BIO-RAD) system. At least three gels with DNA products obtained in each of three independent iPCR assays were analyzed. *In vitro* DNA amplification in these iPCR assays was based on equation:

(7) $$\begin{array}{r} {P=P_{0}\left(1+E\right)}^{C}, \end{array}$$

where *P* and *P*_0_ are, respectively, the amount of amplified linear pDNA product and the initial amount of template pDNA in the gDNA used for the amplification reaction; *E* is the amplification efficiency, and *C* is the number of cycles. Irrespective of the gDNA concentration used, the ratio between *P* and *P*_0_ is kept constant for a given *C* provided there is no exhaustion of the primers and dNTPs required for DNA synthesis (and hence *E* is kept constant). We then confirmed that the employed iPCR conditions fulfilled this requirement for gDNA concentrations ranging from half to twice that used for the analysis of the kinetics of plasmid repopulation.

On the other hand, we have defined the relative plasmid amplification occurring in the transformants during the time interval *t*_*i*_-*t*_*j*_ as the ratio between the relative numbers of plasmid molecules (with respect to the total gDNA) at times *t*_*j*_ and *t*_*i*_, which can be estimated from the intensity of the bands corresponding to the linear pDNA products obtained in the iPCR assays using the same amount of gDNA extracted at different times after transformation. Considering that, in the time interval *t*_*i*_ to *t*_*j*_, the plasmid replication rate (i.e., the ratio of pDNA duplications, *D*_*P*_, to gDNA duplications, *D*_*C*_,) has a value of *R*, the relative plasmid amplification in this interval is given by the following equation:

(8) $$\begin{array}{r} Pl_{t_{j}}/Pl_{t_{i}}=\;2^{D_{P}}/2^{D_{C}}\;{=2}^{\left(RD_{c}-D_{c}\right)}=2^{D_{c}\left(R-1\right)}, \end{array}$$

where *Pl*_*t*_*j*__ and *Pl*_*t*_*i*__ are the relative intensities of the amplified pDNA products at times *t*_*j*_ and *t*_*i*_, respectively. Equation (8) can be converted into a linear function by taking logarithms:

(9) $$\begin{array}{r} log\left(Pl_{t_{j}}/Pl_{t_{i}}\right)=\;D_{C}\left(R-1\right)log2, \end{array}$$

therefore, the *R* value in the time interval *t*_*i*_ to *t*_*j*_ was calculated from the equation:

(10) $$\begin{array}{r} R=\left(log\left(Pl_{t_{j}}/Pl_{t_{i}}\right)/D_{c}log2\right)+1 \end{array}$$

### Statistical analysis

ANOVA was run to determine whether experimental Q ratios differed among groups of staphylococcal strains (*p*-values < 0.05 were considered significant).

## Results

### The presence of the cop repressor protein of R1 or pMV158 in the recipient cell decreases the frequency and/or the velocity of appearance of colonies transformed with the cognate plasmid

To know whether an initially unrepressed transcription of the essential *rep* gene is required for successful establishment of plasmids R1 and pMV158 in a new cell, we compared the efficiencies with which recipient cells that contain or lack the Cop transcriptional repressor of either plasmid were transformed in parallel with plasmids harboring the cognate replicon or an unrelated replicon. It is worth noting that the incoming plasmids carry their own *cop* genes, and hence these assays aim to analyze the importance of the recipient cells having the Cop protein already synthesized upon plasmid entrance.

**Figure 2:**
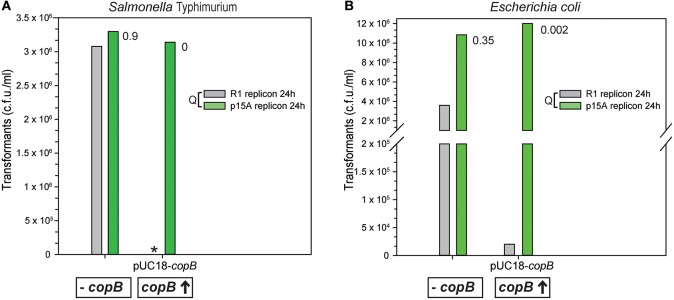
The presence of excess CopB in the recipient cell dramatically and specifically decreases the efficiency of transformation with the R1 replicon. The vertical bar graphs show the number of transformant colonies per ml that appeared after transforming either plasmid-free or pUC18-*copB*-carrying *S*. Typhimurium **(A)** and *E. coli* **(B)** cells with DNAs of plasmids pKN1562 (a mini-R1 derivative) or pACYC184 (harboring the R1-unrelated p15A replicon), both of which are compatible with the pUC18 replicon. The same volumes were plated for all transformed cultures; by plating this volume, 500–1,000 p15A-transformant colonies were counted. Transformant colonies were counted after incubation for 24 h at 30°C. The asterisk in **(A)** indicates the absence of transformants after transforming *S*. Typhimurium SL1344 carrying pUC18-*copB* with pKN1562. The ratio (Q) between the number of transformants per ml obtained with pKN1562 and that obtained with pACYC184 is indicated in the graphs on the right of the corresponding vertical bars.

The existence of a specific effect of R1 CopB on the establishment of the cognate plasmid was tested by transforming either plasmid-free or pUC18-*copB*-carrying *E. coli* C600 and *S*. Typhimurium SL1344 (WT) cells with DNAs of plasmids pKN1562 (a mini-R1 derivative) or pACYC184 (harboring the R1-unrelated p15A replicon), both of which are compatible with the pUC18 replicon. Plasmid pUC18-*copB* provides *in trans* a very high dosage of the *copB* gene cloned under control of its own constitutive promoter. When *S*. Typhimurium cells were transformed with pACYC184, similar transformation efficiencies were obtained irrespective of the presence of CopB in the recipient bacteria (Figure 2A). Also, the frequency of transformation of *S*. Typhimurium cells lacking CopB with pKN1562 was basically the same as with the p15A-derivative plasmid (Figure 2A). Contrarily, the presence of CopB most severely impaired the efficiency of transformation with the cognate plasmid containing the R1-replicon, as no transformant colonies appeared within 24 h of incubation of the plates (Figure 2A). The drastic and specific effect of CopB on the establishment of the R1 replicon was also observed in *E. coli*, where the presence of resident pUC18-*copB* reduced by more than two orders of magnitude the number of colonies transformed with pKN162, without affecting the efficiency of transformation with pACYC184 (Figure 2B).

**Figure 3:**
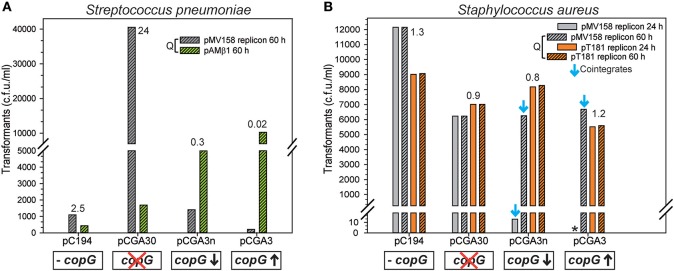
The presence of CopG in the recipient cell decreases the frequency or the velocity of appearance of transformants for the pMV158 replicon in a selective and dosage-dependent manner. **(A)** The vertical bar graph shows the frequency of transformants after transforming *S. pneumoniae* harboring different plasmids with pLS1 (pMV158 replicon) or pAMβ1 (pMV158-unrelated replicon). The resident plasmids provided no *copG* (pC194), inactive *copG* (pCGA30), and medium and high dosages of active *copG* gene (pCAG3n and pCGA3, respectively). The ratio (Q) between the frequency of transformants colonies obtained with pLS1 and that obtained with pAMβ1, counted after 60 h of incubation at 37°C, is indicated on the top of the corresponding vertical bars. **(B)** *S. aureus* cells, harboring the same set of plasmids as described in **(A)**, were transformed with plasmids containing the replicon of either pMV158 or pT181. Vertical bars represent the frequency of transformants colonies counted after 24 h or 60 h of incubation at 37°C. The down facing blue arrow symbol indicates that the pMV158 replicon is only present as a plasmid cointegrate in the transformants. The ratio (Q) between the frequency of transformants colonies obtained with pLS1 and that obtained with pT181*cop608*, counted after 60 h of incubation at 37°C, is indicated on the top of the corresponding vertical bars. The asterisk in **(B)** indicates the absence of transformant colonies appeared within 24 h of incubation. The data presented in this figure summarize the results obtained in typical transformation experiments of *S. pneumoniae* and *S. aureus*. Two additional transformation experiments were performed for each species and the results with respect to the inhibitory effect of CopG on the transformation with the pMV158 replicon were similar to those shown here.

With respect to the pMV158 system, its CopG repressor protein was also shown to significantly impair the establishment of the plasmid, although a differential effect was observed between *S. pneumoniae* and *S. aureus* (Figure 3). In these assays, resident plasmids pC194, pCGA30, pCGA3n, and pCGA3 provided no *copG*, inactive *copG* and medium and high dosages of the autoregulated active *copG* gene, respectively (see Table 2). The specific effect of CopG on the establishment of pMV158 in a new cell was analyzed by comparing the efficiency with which recipient strains containing each of these resident plasmids were transformed in parallel with the pMV158-derivative plasmid (pLS1) and with a pMV158-unrelated plasmid (pAMβ1 in *S. pneumoniae* and pT181-*cop608* in *S. aureus*).

In the pneumococcal host, the plasmids whose establishment was to be analyzed were introduced by natural transformation, a horizontal gene transfer mechanism that requires the development of a transient physiological property named competence. Many different factors have been shown to affect competence (Attaiech et al., 2015), and we actually observed quite different chromosomal transformation frequencies in the various strains used. Namely, the strain harboring pCGA3 showed the highest transformation frequency, followed by the strain containing pCGA3n, next that harboring pCGA30, and finally the strain with the pC194 vector exhibited the lowest competence level (see Material and Methods). The same qualitative trends were observed when analyzing the efficiencies of transformation of the various strains with plasmid pAMβ1 (whose replicon is not repressed by CopG), although in this case quantitatively larger differences were observed among them (Figure 3A). In order to normalize the frequencies of transformation with the pMV158 derivative with respect to the level of competence for plasmid transfer, the ratio (Q) between the transformation efficiencies with the pMV158 derivative and with pAMβ1 was used as parameter. This allowed us to evaluate the specific effect of CopG on the establishment of its cognate replicon. Since the pAMβ1 transformants grew slowly, total transformant colonies were only counted after 60 h of incubation at 37°C, regardless of which plasmid was transferred into the recipient cells. When the void pC194 vector was the resident plasmid, pLS1 yielded 2.5-fold more transformants than pAMβ1. This ratio increased to 24 when the recipient cells contained high-copy-number plasmid pCGA30 providing an inactive *copG* gene. The observed increase in the transformation efficiency is most likely due to facilitation of plasmid transformation by the existence of homology between the plasmid and the genome of the recipient cell, which in this case arises from the presence of a *copG* fragment in both the resident and the entering plasmid. The phenomenon of facilitation has only been reported to occur in natural transformation systems where donor DNA enters the competent cells in a linear ssDNA form (López et al., 1982). In spite of the potential facilitation of the transfer of the pMV158 derivative due to the presence of the entire *copG* gene in the recipient cells, the ratio of pLS1 to pAMβ1 transformant colonies was reduced to 0.3 and to 0.02 when the resident plasmid provided, respectively, medium (pCGA3n) and high (pCGA3) dosages of active *copG* (Figure 3A). Two other independent lots of competent cells of the various strains were transformed in parallel with pLS1 and pAMβ1, and similar Q ratios were obtained in both cases. These results show that the presence of CopG in the recipient cell severely and specifically impairs the success of the establishment of the cognate pMV158 replicon by decreasing the frequency of transformation, as we have also shown to be the case with CopB of the R1 system in both *E. coli* and *S*. Typhimurium (Figure 2).

In the staphylococcal host (Figure 3B), slightly different electrotransformation efficiencies were observed for the various strains, although all pT181-*cop608* transformant colonies appeared within the first 24 h of incubation irrespective of the presence or absence of CopG in the recipient cells. This was also the case for the pLS1 transformant colonies provided that the recipient strains lacked CopG (i.e., when the resident plasmids were pC194 or pCGA30). In contrast, virtually all pLS1 transformant colonies of the recipient strains carrying medium or high dosages of active *copG* could only be detected after 24 h of incubation, and thus they were counted at 60 h after plating. Despite the delay caused by CopG in the growth of the staphylococcal cells transformed with the pMV158 replicon, the ratio of pLS1 to pT181-*cop608* transformant colonies after 60-h incubation was close to 1, regardless of the plasmid resident in the recipient strain. Two more *S. aureus* transformation experiments were performed using the frozen stocks of electrocompetent cells. Although in these experiments the efficiencies of transformation with either plasmid were lower than when freshly-prepared cells were used, the Q ratios remained near constant. The mean Q value for all the strains was 1.1. Analysis of variance (ANOVA) of all experimental Q ratios indicated that there were no significant differences among groups, i.e., the same final frequency of pLS1 transformants was obtained irrespective of the presence of CopG in the recipient cell. Hence, in *S. aureus* CopG seems to specifically impair transformation with the pMV158 replicon by decreasing the velocity of growth, but not the final frequency, of the transformants (Figure 3B).

**Figure 4:**
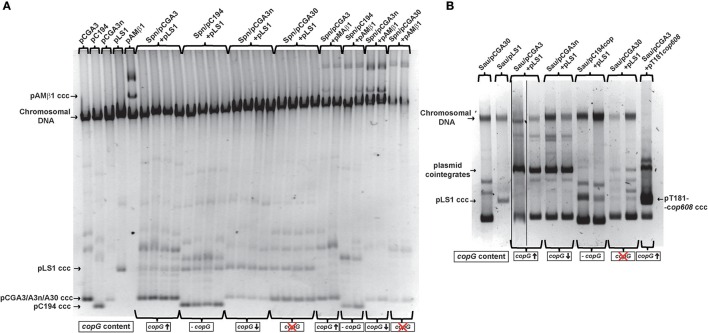
Analysis of the plasmid DNA content of the indicated transformant clones grown under selective pressure for both the resident and the incoming plasmid. **(A)** Pneumococcal cells harboring pCAG3, pC194, pCGA3n, or pCGA30 were transformed with pLS1 and pAMβ1. The gel shows the total DNA content of several transformant clones. Four transformant clones were analyzed for each transformation with pLS1 and only two clones in the case of each transformation with pAMβ1. The combination of recipient strain and incoming plasmid is indicated on the top of the gel. The *copG* gene content of the resident plasmid in the recipient strain is indicated below the gel image. Homoplasmid strains harboring pCGA3, pC194, pCGA3n, pLS1, or pAMβ1 were used as controls. Supercoiled monomeric forms of the five plasmids are indicated in the gel; monomeric forms of pCGA3, pCGA3n, and pCGA30 have a similar electrophoretic mobility. **(B)** Staphylococcal cells harboring pCAG3, pCGA3n, pCGA30, or pC194 were transformed with pLS1 and pT181*cop608*. The gel shows the plasmid DNA content of several transformant clones. Two transformant clones were analyzed for each transformation experiment with pLS1, and one clone resulting from the transformation of *S. aureus*/pCGA3 with pT181*cop608* was also analyzed. The combination of recipient strain and incoming plasmid is indicated on the top of the gel. As in **(A)**, the *copG* content of the recipient strain in indicated below the gel image. Homoplasmid strains harboring pCGA3 or pLS1 were used as controls. Supercoiled monomeric forms of pLS1 and pT181*cop608* are indicated in the gel.

To investigate the basis of the differential effect of CopG on the establishment of the pMV158 replicon in *S. pneumoniae* and *S. aureus*, we analyzed the plasmid content of various transformants of either species grown under selective pressure for both the resident and the incoming plasmid. This analysis was facilitated because the medium or high steady-state copy number of both resident and newly-acquired plasmids in these bacteria allows us to visualize the plasmid bands after electrophoretic separation and staining of DNA minipreps. In *S. pneumoniae*, total crude DNA extracts showed the presence of the expected resident and newly-acquired plasmids in all transformant clones (Figure 4A). When the co-resident plasmid provided no active *copG* (pC194 and pCGA30) or medium dosages of the active gene (pCGA3n), the steady-state copy number of pLS1 in the corresponding transformant clones remained the same as in the homoplasmid situation, whereas a ~35% decrease in the pLS1 copy number was observed if this plasmid coexisted with pCGA3, which provides high dosages of the active *copG* gene. A similar effect of the different dosages of *copG* supplied *in trans* on the steady-state copy number of the pMV158 replicon has been previously reported in transformant clones arising from the reverse transformations (i.e., when pneumococcal cells carrying pLS1 were transformed with the various pC194 derivatives) (del Solar et al., 1995). On the other hand, plasmid DNA minipreps of staphylococcal transformant clones (Figure 4B) only revealed the presence of the two expected plasmids when the resident plasmid was pC194 (no *copG*) or pCGA30 (inactive *copG*). In the latter case, however, an additional slight DNA band appeared that, according to restriction analysis, corresponded to a cointegrate generated by homologous recombination between the resident and the newly-acquired plasmids through the pMV158 DNA region cloned in the resident plasmid (not shown). When the resident plasmid carried an active *copG* gene (pCGA3 or pCGA3n), no separate pLS1 plasmid could be observed and the pMV158 replicon was only present as cointegrate (Figure 4B). It is worth noting that, unlike total DNA extracts from *S. pneumoniae*, the method used to extract the plasmid DNA from *S. aureus* gave random yields and thus could not be employed to estimate the plasmid copy number. Since CopG selectively inhibits the pMV158 replicon, generation of cointegrates with the resident plasmid allows incoming pLS1 to escape from replication inhibition and yet to reach the concentration required to render the host cell resistant to the antibiotic (Tc) with which transformants are selected. According to the results obtained, the exclusive presence of pLS1 as cointegrate occurs in all staphylococcal transformants where an active *copG* gene is provided by the resident plasmid (Figures 3B, 4B), whereas cointegrate formation does not seem to be the strategy used by the equivalent pneumococcal transformants (Figures 3A, 4A), even though cointegration between the incoming and resident plasmids can also occur in this bacterium (Figure S1). This distinct behavior might arise from differences in the frequency with which this Campbell-like recombination takes place in these two bacteria. Cointegrate formation seems to be ultimately responsible for the delayed appearance of the staphylococcal transformant colonies.

To further analyze the role of the Cop proteins in the kinetics of plasmid repopulation, we chose the pMV158 replicon and its pneumococcal host as a model system because of a number of reasons. Importantly, compared to R1, the pMV158 replicon has a higher copy number in both the staphylococcal and the streptococcal host, and hence the amplitude of the plasmid amplification during the establishment phase replication is expected to be also greater, thus increasing the accuracy of the analysis. Moreover, the pneumococcal host of pMV158 was selected because we have a deep knowledge of it and we have set up a higher-frequency transformation protocol. And last but not least, no cointegrates that could mask repopulation of the pMV158 replicon have been observed in this system.

### The presence of the pMV158 CopG repressor protein in the recipient cell results in segregational instability of the incoming pMV158 replicon

**Figure 5:**
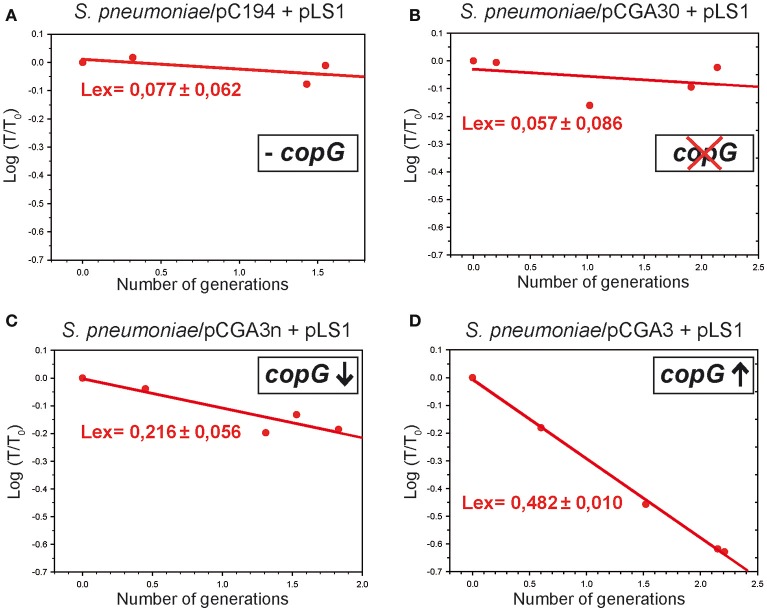
The presence of CopG in the recipient cell unstabilizes the inheritance of the incoming pMV158 replicon in a dosage-dependent manner. The graphs represent the changes in the fraction of transformants retaining the newly-acquired pMV158 replicon (pLS1) when growing for several generations in the absence of selective pressure for the plasmid. The recipient pneumococcal strains lacked *copG* **(A)**, had a truncated version of *copG* **(B)**, or harbored a medium **(C)** or high **(D)** dosage of the active *copG* gene. The experimental loss rate (*L*_*ex*_) of pLS1 was calculated from the slope of the linear regression model of the plot of the experimental values according to Equation (2) (red circles and lines). *T*_0_ and *T* are, respectively, the fractions of transformants *ab initio* and after *n* generations.

As a first approach to study the effect of CopG-mediated transcriptional repression of the essential *repB* gene on the success and velocity of repopulation of the pMV158 replicon, we tested the stability of inheritance of newly-acquired pLS1 in the transformant population of pneumococcal cells carrying null, medium, or high dosage of active *copG* (Figure 5). To this end, we analyzed the change in the numbers of total viable cells and transformants, as well as in the fraction of transformants, at various times after transformation. From these data, the experimental rate of loss of newly-acquired pLS1 from the transformants was calculated (see Material and Methods). The 3-layer *S. pneumoniae* plating method used in these assays (see Material and Methods) allowed the isolation of agar-embedded transformant c.f.u., so that plasmids could repopulate and express their antibiotic resistance determinant before selective pressure was applied.

When CopG-free recipient cells were employed (Figures 5A,B), no loss of pLS1 from the transformants could be inferred, although the fraction of transformants appeared to display a slight decreasing trend. This could be due to the pLS1 burden on the host, which for the steady-state plasmid concentration has been shown to cause an 8–9% increase in the cell doubling time (Hernández-Arriaga et al., 2012), so that the transformants slowly become overgrown by non-transformants.

When the plasmid resident in the recipient cell provided medium dosage of the active *copG* gene (Figure 5C), pLS1 showed unstable inheritance during division of the transformants, with a quite high loss rate per cell and generation (~0.2).

A near-maximal loss rate (~0.5) was determined for newly-acquired pLS1 when the recipient cells contained plasmid pCGA3, which provides high dosages of active *copG* gene (Figure 5D). This maximum segregational instability implies that most frequently pLS1 is inherited by only one of the two daughter cells resulting from division of the transformants. It should be mentioned that, in the pneumococcal host, pLS1 has been shown to be segregationally stable during the steady state, both in the homoplasmid situation (del Solar et al., 1987) and in the presence of recombinant plasmid pCGA3 (del Solar et al., 1995). Hence, plasmid loss in the transformants that harbor extra copies of the active *copG* gene can certainly be ascribed to failures in the establishment phase replication of the pMV158 derivative.

### The CopG repressor protein impairs pMV158 repopulation in the transformants by decreasing the plasmid replication rate

As a further step toward the characterization of the role of CopG during the establishment phase replication of the pMV158 replicon, we have analyzed the kinetics of repopulation of plasmid pLS1*cop*7, a copy-up derivative of pLS1, in pneumococcal cells carrying high dosages of either active or inactive *copG* gene. Compared to pLS1, pLS1*cop7* has a single-point mutation in the *copG* gene, thus encoding a defective CopG protein that leads to a 5-fold increase in the plasmid copy number (del Solar et al., 1990, 1995). The use of pLS1*cop7* in these assays ensures that the effects observed when the resident plasmid carries an active *copG* gene arise from the CopG protein present in the recipient cells and not from that encoded by the incoming plasmid, and also allows determining the replicon repopulation kinetics in the absence of any CopG. Moreover, the use of this copy-up derivative of pMV158 was expected to increase the amplitude of the replicative amplification in the case that repopulation occurred.

**Figure 6:**
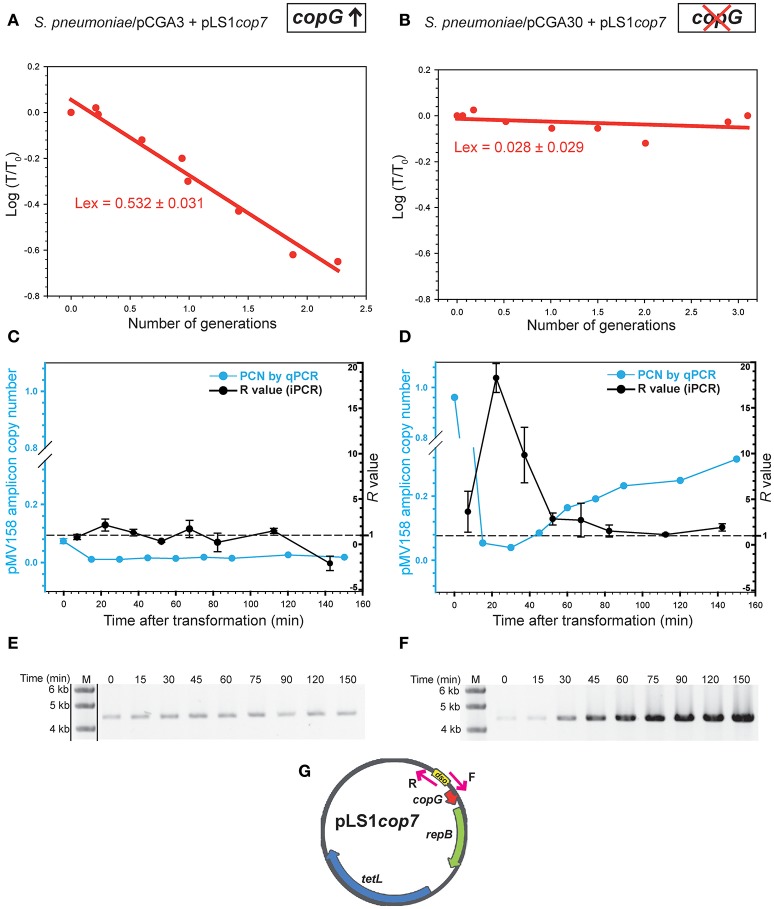
The presence of CopG in the recipient cell impairs repopulation of the pMV158 replicon by decreasing the plasmid replication rate. Changes in the fraction of transformants retaining newly-acquired pLS1*cop7* (a copy-up pMV158 derivative) were analyzed in pneumococcal strains that either harbor a high dosage of active *copG* **(A)** or a truncated version of *copG* **(B)**. The experimental loss rate (*L*_*ex*_) of pLS1*cop7* was calculated from the slope of the linear regression model of the plot of the experimental values according to Equation (2) (red circles and lines). *T*_0_ and *T* are, respectively, the fractions of transformants *ab initio* and after *n* generations. The graphs in **(C,D)** show the impact of CopG on the kinetics of pLS1*cop7* repopulation. A qPCR approach was used to calculate the variation in the copy number of a specific amplicon of the incoming pLS1*cop7* plasmid relative to the chromosome during the growth of the total bacterial population containing pCGA3 **(C)** or pCGA30 **(D)** as resident plasmid for 150 min after transformation (left y-axis; blue circle and lines). The pLS1*cop7* replication rate (*R* value), defined as the ratio of plasmid to gDNA duplications, was calculated at different time intervals following transformation of pneumococcal cells harboring pCGA3 **(C)** or pCGA30 **(D)**. Determination of *R* was based on the iPCR data of the *in vivo* plasmid amplification (black circles and lines) and its value (right y-axis) was calculated according to Equation (10). Discontinuous horizontal line in graphs of **(C,D)** denotes an *R* value of 1, which characterizes the steady-state plasmid replication. The mean (symbols) and standard deviation (error bars) of all the experimental points in the graphs of **(C,D)** are displayed. Panels **(E,F)** show the iPCR analysis of the gDNA samples obtained at the indicated times after transformation of pneumococcal cells carrying pCGA3 and pCGA30, respectively, with pLS1*cop7*. iPCR assays were carried out by using a pair of divergent primers specific for the pMV158 replicon (Table 3 and **G**) and the Phusion DNA polymerase. Lane M, DNA molecular weight standard (NZYDNA ladder III; NZYTECH). Note that lanes M are the same in **(E,F)** because, in fact, both images of these panels arise from the same gel. Dividing lines in **(E)** indicate grouping of different parts of the same gel. The original image of the gel used for **(E,F)** composition is shown in Figure S2. A schematic representation of pLS1*cop7* displaying the plasmid regions complementary to the divergent primers is shown in **(G)**. Genes *copG, repB*, and *tetL*, as well as the *dso* region, are indicated.

Plasmid stability assays showed that pLS1*cop7* was inherited rather stably (with no plasmid loss being inferred) when the recipient cells lacked CopG (Figure 6B). In fact, the fraction of transformants was kept almost constant along several generations of cell growth in the absence of selection for the incoming plasmid (Table 4). In contrast, pLS1*cop7* was lost from the CopG-containing transformants at about the maximum possible rate (0.5; Figure 6A), as can be inferred from the transformant fraction being inversely proportional to the number of total cells (Table 4). The results of the segregational stability of newly-acquired pLS1*cop7* coincided with those obtained with incoming pLS1 (Figure 5), thus showing that the amount of CopG provided by the plasmid resident in the recipient cells suffices to severely impair repopulation of the pMV158 replicon.

**Table 4:** Total number of cells, number of generations, and % of transformants at different times after transformation during the growth of the indicated strains in the absence of selective pressure.

**Time after transformation (min)**	***S. pneumoniae*****/pCGA3 + pLS1*****cop7***			***S. pneumoniae*****/pCGA30 + pLS1*****cop7***		
	**Total number of cell generations^a^**	**Total cell count (c.f.u./ml)**	**% of transformants**	**Total number of cell generations^a^**	**Total cell count (c.f.u./ml)**	**% of transformants**
0	0	3.48 × 10^7^	0.89	0	2.32 × 10^7^	0.16
15	0.01	3.52 × 10^7^	0.94	0.07	2.48 × 10^7^	0.16
30	0.23	4.11 × 10^7^	0.87	0.17	2.62 × 10^7^	0.17
45	0.60	5.27 × 10^7^	0.66	0.52	3.32 × 10^7^	0.15
60	0.94	6.72 × 10^7^	0.56	1.01	4.77 × 10^7^	0.14
75	0.99	6.94 × 10^7^	0.45	1.50	6.59 × 10^7^	0.14
90	1.42	9.32 × 10^7^	0.33	2.01	9.37 × 10^7^	0.12
120	1.88	12.85 × 10^7^	0.21	2.89	17.20 × 10^7^	0.15
150	2.26	16.80 × 10^7^	0.19	3.10	19.95 × 10^7^	0.16

a *Total number of cell generations was calculated according to Equation (3)*.

The issue of the impact of CopG on the kinetics of pLS1*cop7* repopulation was first addressed by qPCR assays aimed at quantifying the variation of the number of copies of the incoming plasmid relative to the chromosome during the growth of the total bacterial population in the absence of Tc (Figures 6C,D). When the recipient cells contained resident pCGA30 and hence no functional CopG was provided, an abrupt decline (from ~1 to ~0.04) in the relative copy number of the plasmid amplicon within the total population was observed during the first 30 min of bacterial growth. This decrease was followed by a slower increase in the number of copies of the plasmid amplicon until a value of ~0.32 was reached after 150 min (Table 5B and Figure 6D). On its turn, a smaller decrease (from ~0.07 to ~0.01) followed by a rather constant relative copy number of the plasmid amplicon was observed when the recipient cells provided CopG (Table 5A and Figure 6C). This transient high concentration of the plasmid specific amplicon that decayed very rapidly in the absence of cell division was most unlikely to correspond to intact plasmid molecules, and could rather reflect the features of the mechanism of natural transformation in *S. pneumoniae*. It is worth mentioning that donor plasmid DNA enters the pneumococcal cell as ssDNA segments of both strands, and that two or more fragments of the opposite strands must anneal through overlapping regions at their ends to generate a circular plasmid form with partial dsDNA regions. This is followed by DNA synthesis to reconstruct the intact plasmid molecule. It should also be noted that, in order to have a high frequency of transformation, we added 300–500 plasmid DNA molecules per bacterial cell. Thus, a number of ssDNA molecules that have entered the cell will harbor the plasmid amplicon to be amplified in the qPCR assay, although most of them will never render a reconstructed plasmid molecule and will be degraded by the cellular nucleases instead. The actual content in intact plasmid molecules was then analyzed by iPCR employing gDNA and two divergent primers that specifically annealed to DNA sequences within the pMV158 replicon (Figure 6G). Immediately after transformation with pLS1*cop7*, while the relative amount of the qPCR-detected pMV158 amplicon in the CopG-free recipient cells was maximal (*t* = *0;* Figure 6D), iPCR amplification rendered the faintest band of specific full-length plasmid DNA (Figure 6F). A similar result was observed when the CopG-containing cells were transformed with pLS1*cop7*, although in this case the initial amount of the pMV158 amplicon was much lower (Figures 6C,E). The observed discrepancy between the initial relative amounts of pMV158 amplicon and intact pLS1*cop7* DNA molecules led us to conclude that, in fact, most of the amplicons to be amplified in the qPCR assays were not carried on reconstituted plasmids but on smaller DNA fragments. Hence, the relative copy number of the donor plasmid shortly after entrance is better evaluated from the iPCR assays. At longer times after transformation (30 min and further), when the DNA fragments carrying the amplicon would have declined, an almost perfect match was observed between the intracellular amplifications of the pLS1*cop7* amplicon (evaluated by qPCR) and of the intact plasmid molecules (evaluated by iPCR). This match was observed irrespective of whether the transformants carried or lacked CopG (Tables 5A,B; see also the Discussion). This coincidence allowed us to determine the rate of pLS1*cop7* replication (*R*, defined as the ratio of plasmid to gDNA duplications, and calculated according to Equation 10) along the time, based on the iPCR data of the plasmid amplification which, unlike the qPCR data, were unaffected by the presence of DNA fragments carrying the pMV158 amplicon.

**Table 5:** Kinetics of genomic and plasmid DNA replication during plasmid establishment.

***S. pneumoniae*****/pCGA3** + **pLS1*****cop7***								
**Time after transformation (min)**	**Total number of cell generations^a^**	**Copy number of the plasmid amplicon (qPCR)**	**Time interval**	**Amplification of plasmid amplicon^b^^,^^d^ (qPCR)**	**Amplification of plasmid molecules^c^^,^^d^ (iPCR)**	**R^e^**	**gDNA duplications**	**pDNA duplications**
**A**								
0	0	0.074 ± 0.009						
15	0.01	0.011 ± 0.002	0–15	0.2	1.0	0.8 ± 0.3	0.21	0.17
30	0.23	0.011 ± 0.001	15–30	1.0	1.3	2.1 ± 0.7	0.28	0.60
45	0.60	0.016 ± 0.001	30–45	1.4	1.1	1.3 ± 0.4	0.60	0.80
60	0.94	0.014 ± 0.001	45–60	0.9	0.9	0.3 ± 0.1	0.35	0.12
75	0.99	0.018 ± 0.001	60–75	1.3	1.0	1.7 ± 1.0	0.16	0.27
90	1.42	0.015 ± 0.001	75–90	0.8	0.9	0.2 ± 1.0	0.41	0.09
120	1.88	0.026 ± 0.001	90–120	1.7	1.5	1.5 ± 0.3	0.96	1.43
150	2.26	0.018 ± 0.001	120–150	0.7	0.8	−2.1 ± 0.8	0.06	−0.10
							Total = 3.03	Total = 3.36
***S. pneumoniae*****/pCGA30 + pLS1*****cop7***								
**Time after transformation (min)**	**Total number of cell generations^a^**	**Copy number of the plasmid amplicon (qPCR)**	**Time interval**	**Amplification of plasmid amplicon^b^^,^^d^ (qPCR)**	**Amplification of plasmid molecules^c^^,^^d^ (iPCR)**	**R^e^**	**gDNA duplications**	**pDNA duplications**
**B**								
0	0	0.960 ± 0.010						
15	0.07	0.054 ± 0.020	0–15	0.1	1.4	3.0 ± 1.7	0.24	0.72
30	0.17	0.040 ± 0.001	15–30	0.7	5.1	14.1 ± 1.2	0.18	2.54
45	0.52	0.085 ± 0.013	30–45	2.1	2.3	7.7 ± 2.3	0.18	1.39
60	1.01	0.164 ± 0.021	45–60	1.9	1.7	2.4 ± 0.5	0.54	1.30
75	1.50	0.191 ± 0.025	60–75	1.2	1.2	2.3 ± 1.4	0.20	0.46
90	2.01	0.232 ± 0.023	75–90	1.2	1.1	1.4 ± 0.5	0.34	0.48
120	2.89	0.249 ± 0.018	90–120	1.1	1.1	1.1 ± 0.1	1.10	1.22
150	3.10	0.315 ± 0.025	120–150	1.3	1.2	1.7 ± 0.3	0.38	0.65
							Total = 3.16	Total = 8.76

a *Total number of cell generations was calculated according to Equation (3)*.

b,c *The factor by which the ^b^plasmid amplicon or the ^c^plasmid molecules copy number is increased in the indicated time interval*.

d *Amplification factor for a certain time interval can be calculated as the product of the amplification factors of the reference time intervals*.

e *R value was calculated according to Equation (10)*.

Although varied slightly along the time, and even reached a value of ~2 in the interval between 15 and 30 min after transformation, the replication rate (*R*) of pLS1*cop7* in the transformants carrying CopG fluctuated around 1 (Table 5A and Figure 6C). An *R* value of ~1, which was also inferred from the ratio between pDNA and gDNA total duplications (*R* = 1.1; Table 5A), implies that the plasmid replicated at the same average velocity as the gDNA during the time interval analyzed and, hence, that it failed to repopulate.

When no CopG was present, an overall *R* value of ~2.8 (the ratio of pDNA to gDNA total duplications; Table 5B) was found for incoming pLS1*cop7* during the entire 150-min interval analyzed, although the plasmid replication rate varied substantially along the time (Figure 6D and Table 5B). Repopulation of pLS1*cop*7 mainly occurred during the first 45 min after completion of transformation, with a peak in the plasmid replication rate in the interval between 15 and 30 min (Figure 6D and Table 5B). Afterwards, the plasmid replication rate decreased asymptotically to 1, indicating that some repopulation still occurred at these longer times. There seemed to be also a small increase in the duplication rate of the pMV158 replicon when bacterial growth slowed down (in the 120–150 min interval), which would be also reflected in an increase of the plasmid copy number (Figure 6D and Table 5B). Whether this could indicate an increase of the pMV158 copy number during the stationary growth phase of its host is a potential matter for future investigation.

## Discussion

In this work we have analyzed for the first time the role played by the Cop regulatory loops of R1 and pMV158 in plasmid establishment. The establishment phase replication, which amplifies the plasmid from an initially low concentration to the steady-state copy number, is a crucial process in the biology of naturally transferable plasmids that may importantly affect the success of their colonization and spreading. Nevertheless, plasmid replication during the establishment phase has been much less studied than the steady-state replication. In a pioneer work by Highlander and Novick (1987) the kinetics of repopulation of various pT181 derivatives that carried or lacked a functional asRNA control system were analyzed in *S. aureus* by determining the replication rates and copy numbers of the plasmids after radioactive *in vivo* labeling of total gDNA. A bit later, the establishment phase replication of ColE1 was studied by using Southern blot to determine the number of phasmids containing the plasmid replicon per *E. coli* cell as a function of time after infection (Merlin and Polisky, 1993). It was found that a phasmid containing an up mutation in the RNA II primer promoter replicated at a 15-fold faster rate than the wild type, thus early highlighting the importance of rapid synthesis of the essential RNA II in ColE1 plasmid establishment. So far, however, the scarce characterized examples of repopulation have missed out the analysis of the effect on plasmid establishment of transcriptional repressors that either exert an auxiliary role or act synergistically with an antisense RNA in controlling the steady-state replication. As a first approach to address this analysis, we have investigated whether the presence of the R1 or pMV158 Cop repressor protein in the recipient cell affects the frequency and velocity of appearance of the plasmid transformant colonies. Moreover, by taking pMV158 and its pneumococcal host as a model system, we have developed a new approach for evaluating the kinetics of plasmid repopulation that is based on the estimation of the plasmid loss rate in transformants and on the use of non-radioactive highly sensitive qPCR and iPCR methods.

The results of the frequencies of transformation of different bacterial species with the R1 or the pMV158 replicons show that, when supplied in the recipient cell, CopB from R1 and CopG from pMV158 severely and selectively impair the establishment of their cognate plasmids. Actually, a dramatic decrease in the efficiency of transformation of *E. coli* or *S*. Typhimurium with the R1 replicon, and of *S. pneumoniae* with the pMV158 replicon can be observed when the cognate Cop repressor is present in the recipient cells (Figures 2, 3A). On its turn, in *S. aureus* cointegration with the resident plasmid allows the incoming pMV158 derivative to overcome the CopG-mediated inhibition of its replicon, so that only a delay in the appearance of the transformant colonies, but not a decrease in the final frequency of them, is observed in this bacterium. As seen for the pMV158 replicon/pneumococcal host system, inhibition of plasmid establishment requires the presence of an active *copG* gene and depends on the dosage of this gene (Figure 3A). Since the R1 and pMV158 Cop proteins repress transcription of the respective plasmid *rep* gene from a strong promoter, the requirement of fully unrepressed expression of the essential *rep* genes for the successful establishment of these replicons can be inferred from the results of the transformation experiments performed in this work. The coincidence between the impairing effects of preexisting CopB and CopG on the efficiency of establishment of their cognate replicon in a new host leads us to think that the Cop repressor-mediated blockage of plasmid repopulation observed in the pMV158/pneumococcal host system can be extrapolated to R1 entering its *Enterobacteriaceae* host.

**Figure 7:**
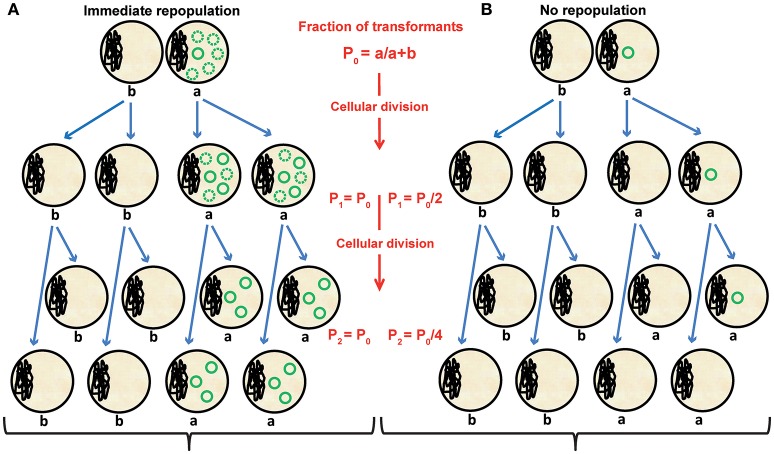
Effect of the velocity of plasmid repopulation on the segregational stability of an incoming plasmid. Two extreme theoretical cases of immediate **(A)** or null **(B)** plasmid repopulation are displayed. These models assume that the plasmid does not burden the host cell. We defined “P_*n*_” as the fraction of transformants after *n* generations; “a” is the initial number of transformants, and “b” is the number of non-transformed cells within the total bacterial population. In the case that immediate repopulation occurs **(A)**, the steady-state plasmid copy number is reached before division of the transformants, and the plasmid is stably inherited to both daughter cells. Dotted circles in **(A)** represent the number of new plasmid molecules generated in each cell generation. If no repopulation occurs **(B)**, the single, unreplicated plasmid copy is inherited by only one of the two daughter cells resulting from division of the transformants, giving rise to maximal segregational instability.

As shown in Figure 7, unsuccessful repopulation of the incoming plasmid may lead to unstable inheritance of the plasmid during division of the transformed cells. The rates at which the pMV158 derivatives (wild-type or copy-up mutant plasmids) are lost during the culture of pneumococcal transformants carrying or lacking a high dosage of autoregulated active *copG* gene match quite well the two extreme theoretical cases of failure or immediate plasmid repopulation, respectively (Figure 7). In fact, a loss rate close to the maximum value (0.5) was found in the first case whereas no significant loss was observed in the second one (Figures 5A,B,D, 6A,B). When a medium dosage of autoregulated *copG* gene is provided by the recipient cell, an intermediate rate of loss of the pMV158 replicon was observed (Figure 5C). Actually, these results indicate that the presence of a functional *copG* gene in the recipient cell causes a dosage-dependent impairment in the pMV158-replicon repopulation that leads to the unstable inheritance of the underpopulated plasmid.

The kinetics of repopulation of the pMV158 replicon, and the effect of the CopG repressor protein on it, have been studied by qPCR and iPCR using total gDNA, prepared at different times after transformation, as template. Irrespective of whether the pneumococcal cells harbor or not the *copG* gene, replication of the gDNA (consisting mainly of chromosomal DNA) appears to begin earlier than cellular division after the 30 to 37°C shift that follows the transformation step (Table 5). The estimated number of total gDNA duplications in the 150-min interval is about three for both kinds of recipient cells, a value that almost equals the number of total cell generations in the case of the recipient cells containing the inactive *copG* gene, whereas it is somewhat higher than the value obtained when the recipient cells contain a functional *copG* gene (Table 5).

In the absence of CopG, the highest repopulation rate occurs during the first 45 min after the entrance of the plasmid DNA in the cell, with a peak in the interval between 15 and 30 min (Figure 6D and Table 5B). According to the results obtained by qPCR (Table 5B), and taking also into account the fraction of transformants (Table 4), the average copy number of pLS1*cop7* in the transformant cells is estimated to be about 200 per chromosome equivalent at 150 min after plasmid transfer. This value coincides with the reported steady-state copy number of the copy-up pMV158 derivative (Table 2), indicating that repopulation has been accomplished by this time. Moreover, no overshoot of the steady-state copy number was observed and instead this value was asymptotically approached (Figures 6D,F). Overall, about nine duplications of the pMV158 derivative took place within the transformants harboring an inactive *copG* gene in the 150-min time interval during which the gDNA duplicated around three times (Table 5B). This represents a 50- to 60-fold relative amplification of the plasmid DNA (see Equation 8), which can also be determined from the amplification factor of the plasmid copy number (Table 5B).

In the presence of CopG, the relative average plasmid copy number in the total bacterial population is kept near constant, as shown by the results from both the qPCR and the iPCR assays (Figure 6C,E and Table 5A). This is also consistent with an overall plasmid replication rate (*R*) of 1.1 along the entire 150-min time period after transformation (Table 5A), which means that, on average, the incoming pMV158 replicon underwent about the same number of duplications as the gDNA. An *R* value of around 1, which in fact characterizes the steady-state replication of any plasmid, demonstrates the unsuccessful repopulation of the pMV158 replicon when CopG is present in the recipient cell. However, based on the maximum possible value of plasmid loss rate that is observed (Figure 6A), a total absence of plasmid replication (*R* ~0 at any time interval) should have been expected instead (Figure 7B). We could envisage two potential explanations for this. One possibility is that in most cells the plasmid replicates at approximately the same rate as the chromosome, thus giving rise to cells containing at least two plasmid copies at division; if so, the observed loss rate would require that the two sister plasmid molecules tend to segregate together into the same daughter cell. An alternative explanation is that plasmid replication manages to evade inhibition by CopG in a small fraction of the transformants so that repopulation to the steady-state plasmid copy number is achieved only in these cells, and hence the maximum value of plasmid loss rate (0.5) would not be significantly altered. Be that as it may, according to the qPCR assays the relative average plasmid copy number in the total bacterial population increases ~1.65-fold during the time interval between 15 min (when the plasmid DNA fragments are considered to be negligible) and 150 min after transformation (Table 5A). When corrected for the fraction of transformants (Table 4), the average plasmid copy number per chromosome in the sub-population of transformed cells is shown to increase from ~1.2 to ~9.5 between 15 min and 150 min after transformation, although at the latter time the plasmid copies might not be evenly distributed among the transformants, as discussed above. Moreover, once the transformant clones are selected for the incoming plasmid, pLS1*cop7* seems to be “forced” to repopulate until reaching the steady-state relative copy number (~33) that is observed in the heteroplasmid situation (Figure S1).

It should be noted that we have obtained similar negative effects on plasmid establishment due to the presence of Cop proteins in the recipient cells when repopulation followed either of two distinct horizontal transfer mechanisms, namely a natural transformation or an electrically-induced transfer. Although the R1 and pMV158 derivatives used in this work did not allow the analysis of plasmid repopulation following conjugative transfer, the significance of fully unrepressed transcription of their essential *rep* genes can most likely be extrapolated to any process implying the establishment phase replication. Establishment phase repopulation is a pivotal step within the process of horizontal plasmid spread by which a unique (or very few) intact dsDNA molecule of the donor plasmid is amplified to the steady-state characteristic copy number. Depending on the transfer mechanism employed, one full-length circular dsDNA plasmid copy can enter directly the cell (electroporation), can be reconstructed from overlapping ssDNA fragments of both strands (pneumococcal natural transformation), or can be generated by synthesis of the DNA strand complementary to the one that was transferred by conjugation. Nevertheless, plasmid repopulation (and its Cop-mediated inhibition) only depends on the replicon function and is independent of the mechanism used for plasmid transfer. In this sense, it is worth noting that similar replication kinetics were observed for the repopulation of pT181 derivatives in *S. aureus* irrespective of whether the initial low plasmid copy number was achieved by shutoff of the replication of thermosensitive mutants or by introduction of the plasmid DNA into the bacterial cells through high-frequency (50%) transduction (Highlander and Novick, 1987).

Carrying an emergency mechanism that enables strong expression of the essential *rep* gene allows rapid and successful repopulation, which can benefit especially those plasmids whose lifestyle includes colonization of new hosts. This is in fact the case, among others, of conjugative or mobilizable plasmids R1, pIP501, and pMV185, all of which harbor a strong promoter directing transcription of the *rep* gene that is subjected to repression by a plasmid-encoded Cop protein. Although a crucial role in plasmid establishment has been proposed for the Cop regulatory loops of these plasmids, no empirical demonstration of it had been reported so far (Nordström and Nordström, 1985; del Solar et al., 1990; del Solar and Espinosa, 2000; Olsson et al., 2004; Brantl, 2014). We think that the requisite, observed in the present work, of fully unrepressed *rep* transcription for the successful establishment of R1 and pMV158 can be extended to other plasmids encoding a similar Cop regulatory loop.

Understanding the role of the Cop regulatory loop that switches on/off transcription of the essential *r*ep gene may help the design and development of new strategies to control spreading of undesirable plasmids among bacterial populations or to prevent transfer of a specific plasmid to a potential host in mating experiments with multiple plasmids.

## Author contributions

RD-O conceived the idea of bringing together the independent observations obtained with the R1 and pMV158 plasmid systems to prepare a joining article about the role of Cop repressors on plasmid establishment. RD-O and IM-C designed and performed the transformation experiments with the R1 replicon. LL performed the transformation experiments with the pMV158 replicon. JR-M carried out the experiments of the kinetics of pMV158 repopulation and the analysis of plasmid stability in the transformants, wrote the Material and Methods section and also prepared most of the figures. GdS designed the experimental approach and the formulation of the kinetics of repopulation and plasmid stability analyses, participated in the experiments with the pMV158 system and wrote most of the manuscript. All the authors discussed the results and corrected the entire manuscript.

### Conflict of interest statement

The authors declare that the research was conducted in the absence of any commercial or financial relationships that could be construed as a potential conflict of interest.
