# Supplementary material for: Successful Establishment of Plasmids R1 and pMV158 in a New Host Requires the Relief of the Transcriptional Repression of Their Essential rep Genes
Source: Front Microbiol. 2017 Dec 1;8:2367. doi: 10.3389/fmicb.2017.02367 (PMC5717011; doi:10.3389/fmicb.2017.02367)
Supplement: Supplementary file 1 [file DataSheet1.DOCX]

Supplementary Material

**Successful establishment of plasmids R1 and pMV158 in a new host requires the relief of the transcriptional repression of their essential *rep* genes**

**José Ángel Ruiz-Masó, Luis Miguel Luengo, Inmaculada Moreno-Córdoba, Ramón Díaz-Orejas, Gloria del Solar***

Molecular Microbiology and Infection Biology Department. Centro de Investigaciones Biológicas, Consejo Superior de Investigaciones Científicas, Madrid, 28040, Spain.

*** Correspondence:** Gloria del Solar**.** [gdelsolar@cib.csic.es](mailto:gdelsolar@cib.csic.es)

**
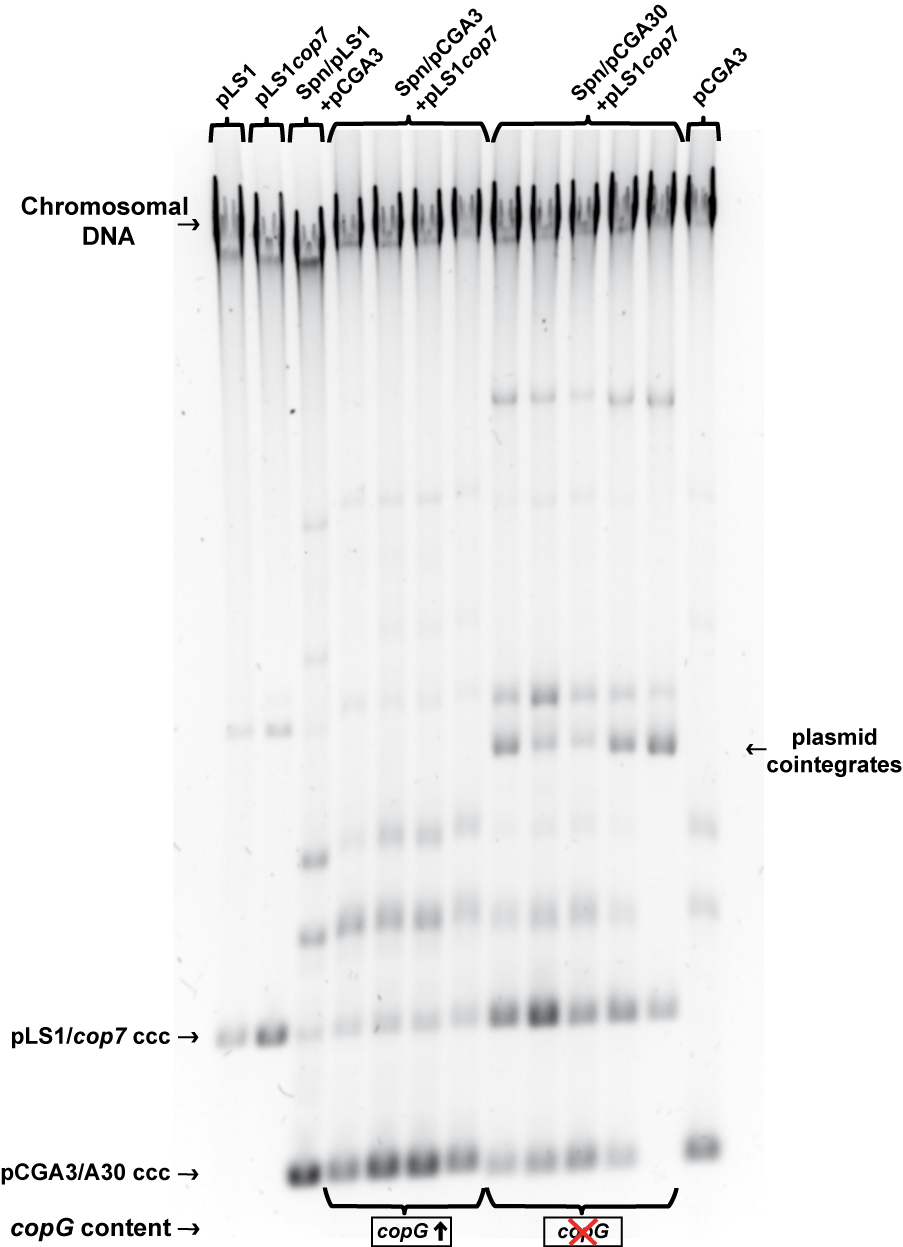
**

**Figure S1. Analysis of the steady-state pLS1*cop7* copy number in pneumococcal cells supplying or not a high dosage of active *copG* gene *in trans*.** Pneumococcal cells harboring pCAG3 or pCGA30 were transformed with pLS1*cop7*, and transformants were selected for both incoming and resident plasmids. The gel shows the total DNA content of several transformant clones from each transformation. The combination of recipient strain (with the resident plasmid indicated) and incoming plasmid is shown on top of the gel. Homoplasmid strains harboring pLS1, pLS1*cop7* and pCGA3 were used as controls. A transformant clone that arose from the transformation of pneumococcal cells harboring pLS1 with pCGA3 was also used as a control. Supercoiled monomeric forms of pLS1/pLS1*cop7* and pCGA3/pCGA30, as well as the position of the plasmid cointegrates, are indicated in the gel. The average PCN of pLS1*cop7* in the strain that also carries pCGA3 was 38.2 ± 2.3, as estimated from the analysis of these DNA preparations according to the protocol given in Material and Methods. The PCN of pLS1*cop7* in the strain also harboring pCGA30 could not be accurately estimated due to the presence of different fractions of the pMV158-derivative as a cointegrated plasmid, which can replicate from the pC194 replicon, thus escaping from CopG control.

**
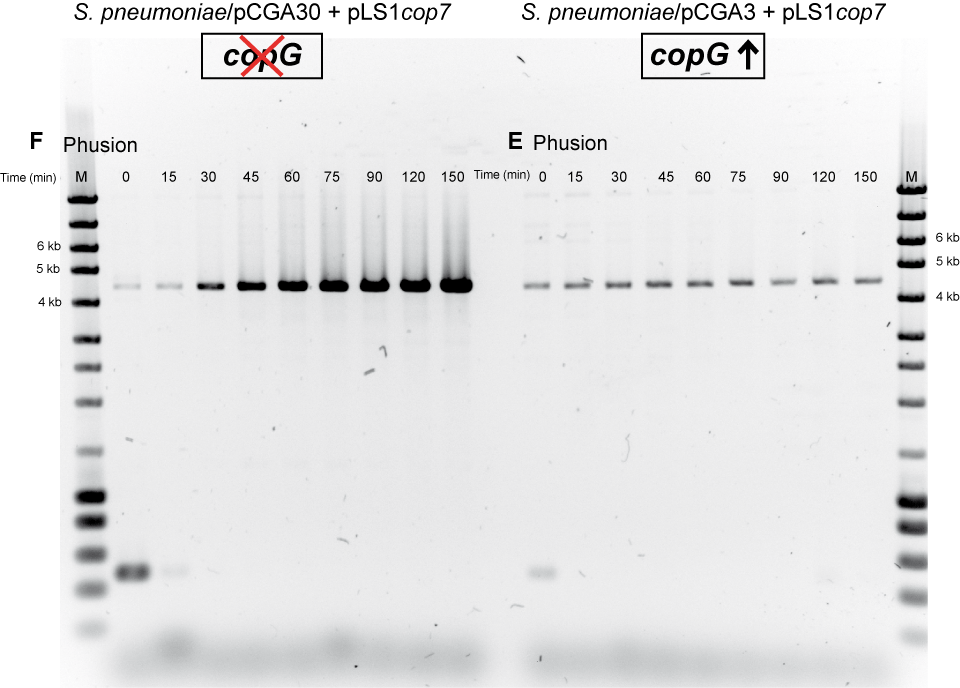
**

**Figure S2. iPCR analysis of the gDNA samples obtained after transformation of pneumococcal cells carrying pCGA3 and pCGA30, respectively, with pLS1*cop7*.** The figure shows the original gel displayed in panels **(E)** and **(F)** of **Figure 6**. As stated in the main text, amplified DNA fragments obtained from gDNA samples by using a pair of divergent primers specific for the pMV158 replicon (Table 2) and the Phusion polymerase were analyzed on agarose gels. The gel lanes used to prepare panels **6E** (cells carrying pCGA3 + pLS1*cop7*) and **6F** (cells carrying pCGA30 + pLS1*cop7*) are indicated by the same letter in the original gels. Lane M, DNA molecular weight standard (NZYDNA ladder III; NZYTECH).
